# Lectures on the Nervous System and Its Diseases

**Published:** 1836-07-01

**Authors:** 


### LECTURES ON THE NERVOUS SYSTEM AND ITS DISEASES. By *Marshall Hall,* M.D. &c. Octavo. April, 1836.

As it would be impossible to give a review or analysis of the volume of Lectures just published by Dr. Hall, we shall only extract the following critical observations on Dr. Hugh Ley's work, reviewed in the present Number of this Journal.

" It has been recently attempted, by Dr. Hugh, Ley, to found the pathology of this interesting disease upon observations, such as that adduced by Dr. Merriman; but I think, unsuccessfully.

*In the first place,* as far as my memory and judgment serve me, the cases adduced, to suport this view, are not cases in point, but, in reality, cases of other diseases.

*Secondly,* supposing pressure upon the pneumogastric to exist, it would induce totally different phenomena from those actually observed in this disease; and it would not explain the *series* of phenomena which actually occur in it: for,

*Firstly :* Such pressure would induce simple *paralysis.*

This would, in the first place, affect the recurrent nerve, and the dilator muscles of the larynx ; it would induce a partial but *constant* closure of that orifice —a permanent state of dyspnœa, such as occurred in the experiments of Legallois, or such as is observed to be excited in horses, affected with '*cornage*' or *roaring,* as described by M. Dupuy, in his treatise ' De la Fluxion Periodique,' 1829, p. 117, &c.

Secondly, it would induce paralysis of the inferior portion of the pneumogastric, with congestion in the lung or lungs, and the well-known effects upon the stomach, of the division of this nerve.

*Secondly :* The disease in question, on the contrary, variously designated '*peculiar convulsion,*' '*spasm of the glottis,*' &c. is obviously a *part* of a more general *spasmodic* affection, and frequently, indeed most frequently, comes on in the midst of the first *sleep,* in the most *sudden* manner, receding equally *suddenly,* to return, perhaps, as before, after various intervals of days, weeks, or even months. Very unlike paralysis from *any* cause!

*Thirdly :* It not unfrequently involves, or accompanies, as I have said, *other* affections, *indisputably spasmodic,* as distortion of the face, strabismus, contraction of the thumbs to the palms of the hands—of the wrists, feet, toes—general convulsions ! sudden dissolution ! a series of phenomena totally unallied to paralysis.

*Fourthly :* Indeed, the larynx is sometimes *absolutely closed*—an effect which *paralysis* of the recurrent nerve, and of the dilator muscles, *cannot* effect.

*Fifthly :* Paralysis from the pressure of diseased glands would be a far *less curable* disease, a far *less variable* disease, a far *less suddenly fatal* disease, than the croup-like convulsion.

*Sixthly :* Almost all recent cases are at once relieved by attention to three or four things : viz. the state, 1, of the *teeth*—2, of the *diet ;*—3, of the *bowels ;* and, 4, by change of *air ;*—they are as obviously produced or reproduced by the agency of errors in one or more of these.

*Seventhly :* In fact, the croup-like convulsion is a *spasmodic* disease, excited by causes situated in the nervous centres, or eccentrically from them ; in a case of spina bifida, already mentioned, a croupy and convulsive inspiration was induced by gentle pressure on the spinal tumor; in cases from teething, the attack has

---

ment in acute rheumatism. This favourable opinion is, therefore to be considered as on the authority of the individual Reviewer only.—J. J.

been induced and removed many times, by *teething,* and by freely *lancing the teeth;* by crudities, and by emetics and purgatives; by change of air, &c.

*Eighthly:* There is a series of facts which prove the connexion of this disease with other forms of convulsions in children, and with epilepsy in the adult subject.

*Ninthly:* In protracted cases, congestion and effusions within the head occur as *effects* of this disease.

*Lastly:* Innumerable cases of undoubted croup-like convulsions have occurred, in which no enlarged glands could be detected in any part of the course of the pneumo-gastric nerve.

But if the contiguity of enlarged glands with the pneumo-gastric have any concern, in any case, in causing this disease, I believe the action is one totally different from that assigned, and not suspected, by the author of this opinion. It is obviously an action upon this nerve, as an *incident, excitor* nerve, and not as a mere *motor,* or *muscular,* nerve.

I must here detail an experiment upon the pneumogastric made by Mr. Broughton, but hitherto unapplied to any question in physiology or pathology. The pneumogastric was laid bare in a donkey, and pinched *continuously* by the forceps; the animal made a sudden act of inspiration and of deglutition. The nerve was divided: the *upper* or *incident* portion of the nerve was pinched with the same effect as before; the lower extremity of the nerve was pinched without any effect.

I may here, also, refer once more to the interesting experiments of M. Dupuy, p. 130, &c. for a similar fact.

In this manner, I conceive, *irritation* of the pneumo-gastric in the neck *may* have induced the croup-like convulsion. *Pressure* upon this nerve, inducing paralysis of its remote extremity, *could not* possibly induce the phenomena in question.

It would be difficult to adduce a more convincing proof of the *pathological* and *practical* importance of the views of the nervous system, which I am laying before you.

I venture to *suggest* another view of this matter as nearer the truth: viz. that this disease is induced through the *fifth* pair of nerves in *teething,* the *pneumo-gastric* in indigestion, and *spinal* nerves in constipation, as parts of the excito-motory system. The view itself *points* to the most useful and efficient *remedies,* and this is highly important: it points to the teeth, indigestion, and constipation, as *causes,* and to the well-known means of removing them; it points to the important objects involved in change of air, mental quiet, &c.

If, instead of the popular remedy (the warm bath), the *gum-lancet,* and the full warm water *enema,* were *instantly* administered, many little patients would be saved from the effects of this terrible disease. The diet should be barley-water only.

The respiration is actually arrested by closure of the larynx, there are forcible expiratory efforts only, or principally, in the actual convulsion. This need scarcely be described: the eyes are distorted from their axes; the face is drawn into horrible forms; the mouth is filled with foam; the body and the limbs are variously and shockingly convulsed. The countenance is livid with venous blood, affording an index to the condition of the brain. There is perfect coma, sometimes long-continued. Or there may be sudden dissolution!

Sometimes a more transient and partial convulsive movement occurs, like an electric shock. In one deeply interesting case, such a convulsive affection was sometimes ushered in by a sardonic smile. In other moments the little boy was obviously expecting the shock in alarm.

In another very interesting case there were strangury and tenesmus, symptoms leading to the suspicion of calculus. The lancing of the gums afforded immediate relief.

As in the affection noticed in my last lecture, cerebral disease was described as frequently leading to convulsion; so, in those which I have just mentioned, the convulsion frequently leads eventually to cerebral disease, especially congestion and effusion. The due relation of the diseases of the cerebral and true spinal subdivisions of the nervous system is plainly seen. Hitherto there has been little but confusion in our views, both of the pathology and treatment of these several diseases.

We may now discern that, whilst in the cerebral diseases our remedies were chiefly directed to relieve the morbid condition of the arterial or capillary circulation within the head, in the diseases of the true spinal system, our efforts must be made to remove the cause or causes of these diseases, whilst we guard against their effects; viz. undue venous *congestion* of the cerebrum, and *effusion.*

I need scarcely advert to the erroneous views, and, consequently, erroneous mode of treatment, of this affection, of those authors who have considered it as originally an affection of the encephalon. Cause has been mistaken for effect, and effect for cause. The effusion, for example, which is the effect of the previous convulsive struggles, has been considered as their exciting cause. The whole confusion upon this point has arisen from not observing to what subdivision of the nervous system the *first* symptoms belong. I quite agree with Dr. Merriman in condemning as useless, or rather as injurious, the indiscriminate and lavish detraction of blood.

The proper mode of treatment comprises the remedies—

1. *Against the Attacks,*
2. *In the Attacks and in the Threatening of the Attack,*
3. *Against their Effects.*

The remedies against the attacks, or the prevention, consists in avoiding all the exciting causes:—dental, gastric, intestinal irritation; passion; vexation; disturbance; interrupted sleep; &c.

The remedies in the threatening of attacks consist in the watchful and prompt repetition of the same treatment;—lancing the gums, relieving the stomach and the bowels. The *sleep,* especially, should be watched; and if there be a sardonic smile, or starting, or other symptoms, the little patient must be *gently* awoke, and the remedies just enumerated should be administered.

After the gum-lancet, I would advise a copious enema of warm water.

If there be great threatening of an attack, I would tickle the fauces, dash cold water on the face, and irritate the nostrils, having the patient placed, as speedily as may be, in the warm bath.

To guard against the effects of the attacks, we may deplete the blood-vessels about the head with cupping or leeches; apply an alcoholic lotion constantly all over the head; or, if the case be urgent, the ice-cap.

In addition to these measures, the secretions must be corrected, mild mercurials being given, perhaps to affect the system; afterwards, change of air is of undoubted efficacy; and a very mild tonic plan may be added with advantage, as minute doses of the sulphate of quinine, of the carbonate of iron, &c. Sponging with *warm* salt and water is also a valuably auxiliary remedy.

It is impossible to misconceive the vast importance of this subject. If any thing could add to this importance, it is the fact that the convulsions of infancy frequently lay the foundation of epileptic attacks in youth or adult age. Sometimes the transition is so gradual and continuous, that the two affections are proved to be obviously the same." 81.

